# Evaluation of the prevalence of sexually transmitted bacterial pathogens in Northern Cyprus by nucleic acid amplification tests, and investigation of the relationship between these pathogens and cervicitis

**DOI:** 10.4274/tjod.galenos.2019.80269

**Published:** 2020-02-28

**Authors:** Onur Güralp, Ayşegül Bostancı, Esra Özerkman Başaran, Meike Schild-Suhren, Barış Kaya

**Affiliations:** 1 Carl von Ossietzky Oldenburg University Faculty of Medicine, Obstetrics and Gynecology, Klinikum AöR, Oldenburg, Germany; 2 Near East University Faculty of Medicine, Department of Molecular Biology and Genetics, Lefkosa-TRNC, Turkey; 3 Near East University Faculty of Medicine, Department of Obstetrics and Gynecology, Nicosia, Northern Cyprus

**Keywords:** Chlamydia, neisseria, trichomonas, mycoplasma, ureaplasma

## Abstract

**Objective:**

To evaluate the prevalence of pathogens, *Chlamydia trachomatis*, *Neisseria gonorrhea* and *Trichomonas vaginalis*, *Mycoplasma hominis*, *Mycoplasma genitalium*, *Ureaplasma urealyticum*, and *Ureaplasma parvum* in women via multiplex-polymerase chain reaction (PCR)-deoxyribonucleic acid (DNA).

**Materials and Methods:**

Cervical swabs of 273 women in reproductive age who underwent gynecologic examination in our outpatient clinic were evaluated using the multiplex-PCR-DNA method. The presence of cervicitis, contraceptive methods, marital status, and the number of partners were evaluated.

**Results:**

One hundred six (39%) of the 273 women had at least one bacterium, 25 women (9.8%) had two bacteria, and three women (1%) had three bacteria. *U. urealyticum* was the most frequently encountered bacterium (13.9%), followed by *M. hominis* (12.8%), *U. parvum* (12.4%), *C. trachomatis* (5.4%), *M. genitalium* (2.9%), *N. gonorrhea* (2.5%), and *T. vaginalis* (0.3%). Bacterial infection was detected more frequently in women aged <25 years, single, who had multiple partners, and clinically diagnosed with cervicitis. The cervicitis rate was 39% in our study. M. genitalium was significantly more frequent in women with cervicitis than in women without cervicitis (5.6 vs. 1.2%, p=0.005). *C. trachomatis* and *N. gonorrhea*, which are often associated with cervicitis, were comparable in women with and without cervicitis.

**Conclusion:**

Women with clinically diagnosed cervicitis or even with a normal-appearing cervix should be tested using multiplex-real-time PCR-nucleic-acidamplification tests on suspicion of such an infection. *M. genitalium* is an emerging bacterial agent for cervicitis along with *C. trachomatis* and *N. gonorrhea*.

**PRECIS:** To identify the possible risk factors for postpartum urinary retention.

## Introduction

Every year, more than 1 million people are infected with sexually transmitted diseases (STDs)^(1)^. *Chlamydia trachomatis, Neisseria gonorrhea *and *Trichomonas vaginalis* are the very well-known sexually transmissible pathogens, whereas *Mycoplasma genitalium *has recently gained importance in the pathogenesis of cervicitis^(2)^. These bacteria are either asymptomatic or present themselves with mild symptoms, which may easily be overlooked^(1)^. These bacterial STDs may lead to tubal infertility and extrauterine pregnancy as well as chronic pelvic pain, which is associated with a severe socioeconomic burden^(1)^. Besides *Mycoplasma hominis *and *Ureaplasma urealyticum,* *Ureaplasma parvum *may be commensally colonized in the cervix. However, some authors suggest that such colonization may be associated with poor obstetric outcome, postpartum sepsis, and neonatal infections^(3)^. The serologic diagnosis or traditional culture media may not be sufficient for diagnosis^(2,4)^. In some cases, the presence of multiple agents makes it even more difficult to diagnose the actual agents^(4)^. For that reason, due to their high sensitivity and specificity for the diagnosis of STDs, as well their ability to diagnose more than one pathogen at once, multiplex real-time polymerase chain reaction (PCR) nucleic acid amplification tests (NAAT) have gained popularity over conventional microbiologic culture methods^(4,5,6)^. In this study, we aimed to evaluate the prevalence of pathogens including *C. trachomatis, N. gonorrhea,* *T. vaginalis*, *M. hominis*, *M. genitalium*, *U. urealyticum *and* U. parvum *in women via multiplex PCR DNA tests, and to assess the role of these bacteria in women with clinically diagnosed cervicitis who were admitted to our outpatient clinic in Near East University for gynecologic examinations.

## Material and Methods

In this study, the cervical swabs of 273 women in reproductive age who were admitted for gynecologic examinations with symptoms of vaginal discharge or who asked for a screening of sexually transmitted infections without any symptoms to the outpatient clinic of Near East University, Department of Obstetrics and Gynecology, between 2014 and 2016, were examined using the multiplex PCR DNA method. The results were retrospectively evaluated. The study was approved by the Ethics Committee of Near East University on March 31^st^, 2016 (number: of 2016/36-266). Informed and signed consent was obtained from all participants. Inclusion criteria were apparently healthy, sexually active women aged >18 years without pelvic pain or fewer, who were not pregnant, and had not received antibiotic recently for a gynecologic infection. Women with pelvic inflammatory disease (PID) were excluded from the study. All women had a gynecologic examination, and the presence of cervicitis, contraceptive methods, marital status, and the number of partners were documented.

Cervicitis was described as the presence of purulent or mucopurulent discharge and/or hyperemic, edematous and friable (bleeding even with a light touch of a cervical swab) cervix^(7,8)^. The cervical swabs from participants were taken by gynecologists using a single-use speculum with the manufacturer’s kits and were sent to the genetic laboratory.

### Nucleic Acid Isolation Procedure

Nucleic acid isolation was performed in accordance with the manufacturer’s instructions (GeneAll RibospinTM vRD). Swab samples were obtained from the cervix and then transferred to the Medical Genetics Laboratory of Near East University Hospital. The following steps were performed: Centrifugation at 5000 rpm for 15 minutes, addition of buffer (VL, 500 µL), incubation for 10 min at 25 °C, addition of buffer (700 µL RB1), and vortexing. Preparation of the spin column. Removal of residual buffer by centrifugation of the mixture at 12,000 g. Addition of nuclease-free H_2_O. Re-centrifugation at over 10,000 g for 60 seconds. The purified nucleic acid was kept at 4 °C for direct analysis and kept at -70 °C for subsequent analysis.

### Polymerase Chain Reaction

PCR was conducted for detecting the STD panel. The fast track diagnostic urethritis plus real-time PCR kit was used for analysis, which examines *C. trachomatis, N. gonorrhea,* *T. vaginalis*, *M. hominis*, *M. genitalium*, *U. urealyticum, *and *U. parvum*. The DNA amplification reactions were performed using Qiagen Rotor-gene Q. After the DNA amplification, the results were interpreted according to the given fluorescence trace of the positive samples. The results were examined using the data supplied by the manufacturer.

### Statistical Analysis

Continuous parametric variables are given as mean and standard deviation. Categorical variables are expressed as number or percentage. T-test or analysis of variance were used for the comparison of parametric variables. Categorical variables were compared using the chi-square (c^2^) test. Statistical calculations were performed using Statistical Package for Social Sciences (SPSS 15.0, Chicago, IL, USA). P<0.05 was accepted as significant.

## Results

A total of 273 women were included in this study. The demographic and clinical features of the patients are given in Table 1. The mean age of the women was 31.03±9.20 years. The study group consisted mainly of married women (70%), with a single partner (85%); 39.5% of the women had cervicitis. One hundred six (39%) of the 273 women had at least one bacterium, 25 women (9.8%) had two bacteria, and 3 women (1%) had three bacteria. Among the 273 women,* U. urealyticum* was the most frequently encountered bacterium in the cervix (13.9%), followed by *M. hominis* (12.8%), *U. parvum* (12.4%), *C. trachomatis* (5.4%), *M. genitalium* (2.9%), *N. gonorrhea *(2.5%), and *T. vaginalis* (0.3%). The infection rates according to the age, marital status, number of partners, the presence of cervicitis, and type of contraceptive method are presented in Table 2. Bacterial infection was detected more frequently in women aged <25 years, those who were single, who had multiple partners, and clinically diagnosed with cervicitis. Bacterial infection was detected less frequently in women who used a condom as a contraceptive method. *M. hominis* was the most commonly seen bacterium in women aged under 25 years. *M. hominis* and *U. urealyticum* were significantly more common in women with multiple partners. The cervicitis rate was 39% among the 273 women in our study. Among women with cervicitis, M. genitalium was significantly more frequent in women with cervicitis than in those without cervicitis (5.6% vs. 1.2% p<0.005).* C. trachomatis *and *N. gonorrhea,* which are often associated with cervicitis, were comparable in women with and without cervicitis. In 55 (33%) of 165 women with no clinical cervicitis, at least one bacterium was detected, and 15 (9%) women had at least one of the bacteria known to be associated with cervicitis, such as *C. trachomatis*, *N. gonorrhea*, *M. genitalium* or *T. vaginalis*. By contrast, among 108 women with clinical cervicitis, the rate of the bacteria known to be associated with cervicitis was 14.9% (9% vs. 14.9%; p=0.133). The rates of simultaneous infections with multiple bacteria were comparable between women with and without cervicitis (9.3% vs. 11.5%, p=0.565).

## Discussion

In our study, 39% of women had at least one bacterium. Among the 273 women, *U. urealyticum* was the most frequently encountered bacterium in the cervix (13.9%), followed by *M. hominis* (12.8%), *U. parvum* (12.4%), which showed a balanced distribution. The detection rate of bacteria was reported to vary between 30.7 and 49% in previous screened populations^(4,5)^. Kim et al.^(5)^ screened 799 Korean women and detected at least one bacterium in 49% of women. Contrary to our study, *U. parvum* was the most frequently (32.5%) found bacterium, followed by *U. urealyticum* (3.5%) and *M. hominis* (1%). Lee et al.^(4)^ screened 304 women and detected bacteria in 36.5%, most frequently *U. urealyticum* (14.5%), followed by *M. hominis *(13.8%). In south Italy, Del Prete et al.^(9)^ screened 1272 women and detected at least one bacterium in 30.7% of women. The most commonly detected bacterium was by far *U. parvum *(25.9%). In our study, we detected the bacterial colonization of *U. parvum *with a rate of 12.4%. Yamazaki et al.^(10)^ reported high detection rates of *U. parvum *as 41.7%. Yamazaki et al.^(10)^ suggested that the high prevalence of the latter two bacteria might be attributed to the region, culture, and tendency to nightlife. Camporiondo et al.^(6)^ performed a screening study in 309 Italian women and detected no *C. trachomatis*, *M. genitalium* or N.* gonorrhea,* but *U. parvum* (28.8%), *M. hominis* (3.9%) and *U. urealyticum* (4.5%). McIver et al.^(11)^ evaluated 175 sexually active Australian women and detected U. parvum (53%), *M. hominis* (7.4%), and *U. urealyticum* (3.4%) in descending order. Simultaneous infection rates with *U. parvum *+ *M. hominis*, *U. urealyticum* + *M. hominis* and *U. urealyticum *+ *U. parvum *were 7.4%, 1.1%, and 2.9%, respectively. In our study *U. urealyticum*, *M. hominis*, and *U. parvum* were among the most commonly detected bacteria in the cervix. They are accepted as genital commensalistic organisms and found in healthy women^(10)^. Routine screening and treatment of the latter three bacteria are controversial^(10)^. Some authors suggest that colonization with *U. urealyticum* and *U. parvum *in high density is associated with non-specific cervicitis^(12)^, whereas others suggest that there is not enough evidence to suggest that these bacteria cause cervicitis or PID^(13)^. However, several studies in pregnant women showed that the presence of these bacteria in amniotic-fluid or membranes might be associated with preterm labor, preterm premature rupture of membranes (PPROM), and neonatal infections^(14,15,16,17,18,19,20,21,22,23,24)^. Abele-Horn et al.^(21)^ showed that *U. urealyticum* was associated with preterm labor. Kataoka et al.^(22)^ evaluated 877 pregnant women under 11 gestational weeks (GW) and detected the prevalence rates of *U. urealyticum, M. hominis*, and *U. parvum* as 52.0%, 11.2%, and 8.7%, respectively. Despite the higher prevalence of U. urealyticum in the latter study, *U. parvum* had a stronger association with late abortion and preterm labor compared with *U. urealyticum*^(22)^. One hundred eighty-four pregnancies complicated with preterm labor and PPROM were evaluated in a prospective study and coinfection with *M. hominis,* and *U. urealyticum *was shown to be associated with poorer pregnancy outcomes compared with infection with *U. urealyticum *alone^(23)^. In another study, vaginal *U. urealyticum* and *U. parvum* colonization were also shown to be associated with chorioamnionitis in pregnancies under 28 GW complicated with PPROM^(24)^. Rumyantseva et al.^(25)^ recently evaluated 1773 women and observed that the isolation rates of *U. parvum* and *M. hominis* in women with bacterial vaginosis were significantly higher in women with altered vaginal microflora compared with women with normal vaginal flora. Chlamydia is known to be the most common STD^(26,27)^. It is one of the major organisms causing cervicitis and PID, even if it is asymptomatic. In our study, the prevalence of *C. trachomatis* was 5.4%, which was more frequent than other sexually transmissible bacteria such as *M. genitalium* (2.9%), *N. gonorrhea* (2.5%) and *T. vaginalis *(0.3%). Chlamydia prevalence, along with the prevalence of other STDs, may vary according to the age, race, region, and socioeconomic status^(26,28)^. The prevalence of chlamydia was reported as 0.6% in Australia^(11)^, 2.6% in the Netherlands^(29)^, 2.3% in China^(30)^, and as high as 14.2% in South Africa^(31)^. The prevalence of chlamydia in the United States of America (USA) was reported to be 4.2% in the general population, but as high as 10% in Mexicans living in the USA (26). In a systematic review and meta-analysis, the prevalence of chlamydia in Europe and developed countries such as Canada, Australia, and New Zealand was reported as 3.0-5.3%^(28)^, which is also concordant with the values in our study. In the present study, the prevalence of gonorrhea was 2.5%. Gonorrhea is the second most common sexually transmitted bacterial infection following chlamydia^(26)^. According to the World Health Organization report in 2012, the global gonorrhea prevalence varies between 0.3% and 1.7%^(1)^. In our study, the prevalence of gonorrhea was significantly over the global average. Nevertheless, the prevalence of STD may vary according to the country or even to the region in the same country. Kim et al.^(5)^ evaluated 799 Korean women and detected no gonorrhea, whereas Lee et al.^(4)^ evaluated 304 Korean women and detected gonorrhea in 3.3% of the screened population, which was even higher than in our study group. Gaydos et al.^(32)^ evaluated a group of 324 women comprising mainly young African-American women in Baltimore, USA, and detected the rate of gonorrhea as 4.6%. The high rates of gonorrhea in our study may be attributed to the presence of nightclubs in our region. In our study, the rate of *M. genitalium* infection was 2.9%. The global *M. genitalium* infection rate is reported as 1-6.2%^(33)^. Nevertheless, prevalence rates as high as 19.2% have also been reported^(32)^. Nowadays, since the widespread use of NAATs in recent years, *M. genitalium* counts as one of the most important bacteria, following *N. gonorrhea* and C*. trachomatis*, causing cervicitis^(34)^. The prevalence of *T. vaginalis* was 0.3% in our study. *T. vaginalis* is the most common non-viral sexually transmissible infection in the USA and may cause urethritis in men and women, and vaginitis and cervicitis in women^(35)^. It is hard to determine the true prevalence of *T. vaginalis* because the feedback is not so efficient as with other STD pathogens. According to the Centers for Disease Control and Prevention, the prevalence of *T. vaginalis* in non-Hispanic women in the USA is 1.8%^(2)^. Moreover, in hitherto literature, prevalences of *T. vaginalis* as low as 0.1% have been reported^(5)^.

### Cervicitis

In our study, the cervicitis rate was 39% among 273 women, which is similar to the 41% among 324 women reported by Gaydos et al.^(32)^. Gaydos et al.^(32)^ detected that *C. trachomatis* and *M. genitalium* were associated with cervicitis; however, only *M. genitalium* had a significant association with cervicitis in multiple regression analysis. In our study, the *M. genitalium* infection rate was significantly elevated in women with clinical cervicitis compared with women without cervicitis, which supports recent data about the importance of *M. genitalium* as an emerging pathogen of cervicitis. The clinical diagnosis of cervicitis is not always suggestive for a sign of bacterial infection. It has been shown that no infectious pathogen is detectable in the majority of women with clinical cervicitis^(36)^. Nevertheless, *N. gonorrhea* and *C. trachomatis* have long since been reported to be the most frequent bacteria causing cervicitis, if a pathogen is detectable^(37,38)^. Along with *N. gonorrhea* and *C. trachomatis*, *M. genitalium*, *T. vaginalis*, and Herpes simplex virus, as well as *Gardnerella vaginalis*, may cause cervicitis^(34)^. In our study, there was no detectable bacterial pathogen with NAAT PCR DNA in 57 of 108 women (52%) with clinical cervicitis. At this point, it should be mentioned that etiologic factors of non-infectious cervicitis include chemical or physical trauma, vaginal douche, idiopathic inflammation of the cervix, and Behçet’ disease^(2)^. In our study, in 55 (33%) of 165 women with no clinical cervicitis at least bacterium was detected, and fifteen (9%) women had at least one of the bacteria known to be associated with cervicitis, such as *C. trachomatis*, *N. gonorrhea*, *M. genitalium* or *T. vaginalis*. By contrast, among 108 women with clinical cervicitis, the rate of the bacteria known to be associated with cervicitis was 14.9%. The coinfection rates in women with and without cervicitis were comparable in our study, concordant with the findings of Gaydos et al.^(g)^. Studies about the detection rates of infectious agents causing vaginitis and cervicitis are summarized in Table 3^(4,5,6,9,11,32,39)^. In our study, it has been shown that multiplex real-time PCR-NAAT is beneficial for the demonstration of cervical bacterial colonization with commensal pathogens including *U. urealyticum*, *M. hominis*, and *U. parvum* or with pathogens that can cause cervicitis such as *C. trachomatis*, *N. gonorrhea*, and *M. genitalium*. The major limitation of these techniques is their high costs^(40)^.

## Conclusion

In our study the prevalence of sexually transmitted bacteria in the Northern Cyprus region was consistent with the literature. *M. genitalium* was detected to be more frequent in women with cervicitis than in women without cervicitis (5.6% vs. 1.2% p<0.005). *C. trachomatis* and *N. gonorrhea*, which are often associated with cervicitis, were comparable in women with and without cervicitis.

According to our study results, women either diagnosed with cervicitis or with a normal-appearing cervix should be tested with multiplex-real-time PCR-NAATs on suspicion of such an infection.

There was no significant difference regarding simultaneous infection with multiple bacteria between women with and without cervicitis.

## Figures and Tables

**Table 1:**
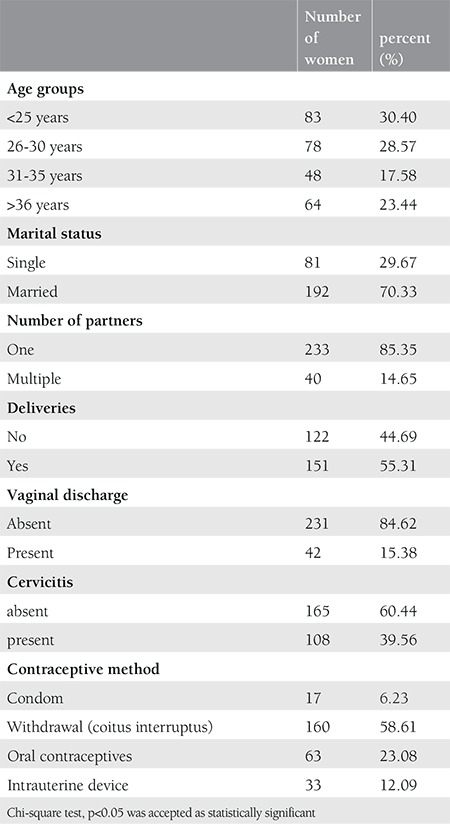
The demographic and clinical features of the patients

**Table 2:**
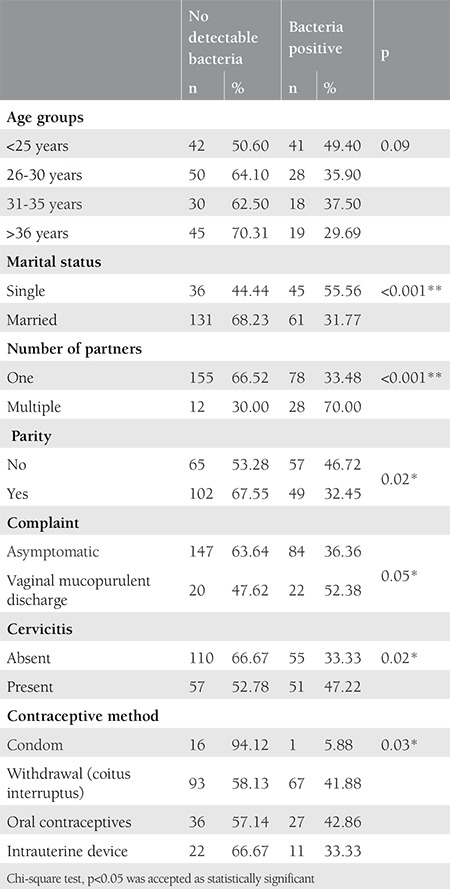
The infection rates according to the age, marital status, number of partners, vaginal mucopurulent discharge, presence of cervicitis and type of contraceptive method

**Table 3:**
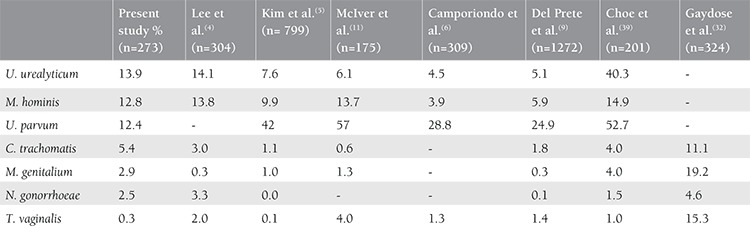
Studies about the detection rates of infectious agents causing vaginitis and cervicitis
